# Regeneration of the Chrystaline Lens

**Published:** 1848-08

**Authors:** 


*Regeneration of the Crystalline Lens.*—M. Stromeyer has shown to the Medical Society of the department of the Upper Rhine, a lens regenerated after the operation of cataract. The patient died four years after having been operated upon. The organ reproduced was not exactly a lens, but a ring, perfectly transparent before its immersion in alcohol. The annular form is attributed by M. Stromeyer to adhesions between the anterior and posterior parietes of the lens, and he puts the question whether these abnormal adhesions, in the hope of renewed occurrence of such a regeneration, could not be prevented.—*Ibid.*

